# Edaravone for Potential Mitigation of Oxidative Stress and Inflammation in Kidney Disease

**DOI:** 10.3390/biom16101490

**Published:** 2026-10-13

**Authors:** Yasmeen K. Mohamed, Liang-Jun Yan

**Affiliations:** Department of Pharmaceutical Sciences, UNT System College of Pharmacy and Health Professions, UNT Health Fort Worth, Fort Worth, TX 76107, USA; yasmeen.mohamed@unthsc.edu

**Keywords:** edaravone, acute kidney injury, chronic kidney disease, oxidative stress, inflammation, fibrosis

## Abstract

Edaravone (EDA) is a synthetic free-radical scavenger FDA-approved for amyotrophic lateral sclerosis (ALS) treatment in the United States and is used clinically for treatment of acute ischemic stroke in Japan and other countries. While its antioxidant activity has been the primary focus of investigation in the mitigation of oxidative-stress-related injury, EDA exhibits anti-inflammatory, anti-fibrotic, anti-apoptotic, and anti-necrotic effects in animal models. These properties make it a potentially attractive therapeutic agent for kidney disease, where oxidative stress and inflammation contribute to renal cell injury, mitochondrial dysfunction, and progression of disease. In this review, we cover the underlying mechanisms of oxidative stress and inflammation in renal injury, and pharmacological and free-radical-scavenging mechanisms of EDA. Preclinical evidence in several kidney diseases, including ischemic/reperfusion injury, nephrotoxin-induced disease, and diabetic kidney disease, has been used to assess EDA for efficacy. In addition, we explore the modulatory roles of key signaling pathways, such as Nrf2 and NF-κB, in protective effects of EDA against oxidative stress, inflammation, mitochondrial dysfunction, apoptosis, necrosis, and fibrosis. Finally, we highlight the potential of EDA as a therapy in future clinical applications for various kidney diseases such as acute kidney injury (AKI) and chronic kidney disease (CKD). It should also be emphasized that as EDA’s renal efficacy evidence described in this review is largely limited to animal studies, robust human clinical data are lacking. Therefore, additional clinical studies are still needed to fully elucidate the efficacy, safety, and benefits of EDA for prevention and treatment of human kidney disease.

## 1. Introduction

The kidneys receive 20–25% of cardiac output in the resting state [1]; thus, there is remarkably high renal blood flow. Each kidney contains roughly one million nephrons, which facilitate the removal and elimination of toxic substances. As such, they are exposed to high volumes of circulating substances in the blood. In particular, the primary site of filtration is the nephron’s glomerulus. The glomerulus filters plasma into Bowman’s space, after which the filtrate moves into the proximal tubules. The proximal tubular epithelial cells have high metabolic demand and accordingly contain a high abundance of mitochondria [2]. The kidneys are the second most mitochondrially enriched organs in the body, after the heart [3]. Proximal tubular epithelial cells are highly exposed to toxins entering the nephron. Accumulation of waste and endogenously generated reactive oxygen species (ROS) increases the risk of toxic injury [4,5].

Kidney disease affects 15% of the adult US population (roughly one in seven people) and is a major healthcare and socioeconomic burden [6]. Acute kidney injury (AKI) and chronic kidney disease (CKD) are common and contribute to significant morbidity and mortality [7,8]. Furthermore, kidney diseases are closely associated with development and progression of well-established comorbidities including cardiovascular disease, cancer, sepsis, and cerebrovascular disorders such as acute ischemic stroke, while diabetes contributes significantly to development and progression of renal disease as a major risk factor [9,10,11,12,13]. Without effective intervention, kidney disease and its common comorbidities are associated with increased mortality, highlighting the need for novel treatments targeting the underlying mechanisms driving pathology.

Exposure to nephrotoxic substances, including heavy metal toxins such as cadmium and mercury [14,15], can occur through food, contaminated water, smoking, heavy pollution, and occupational or other environmental risk factors and may also increase risk of kidney disease [14]. Metabolism of synthetically produced vitamins such as folic acid [16], the oxidized form of naturally occurring folate (vitamin B9), may also have potential adverse effects on renal injury and fibrosis in high doses [16,17,18]. Toxins acquired in these contexts increase vulnerability of the kidneys to damage, as certain toxins can accumulate and concentrate in the renal tissue and tubular fluid. Drug toxicities stemming from use of nephrotoxic agents such as cisplatin, a platinum-containing chemotherapeutic, can also increase risk for kidney dysfunction [19,20].

### 1.1. Kidney Injury and Disease Burden

Globally, AKI affects roughly 13.3 million people. CKD has a prevalence of more than 10%, which equates to more than 800 million people [21,22]. In the US, 90% of those with CKD are unaware of their disease, making it the most underrecognized public health crisis and costing Medicare patients more than $70 billion per year [6]. Conflicting definitions of AKI complicate prevalence estimates, as they can range from <1–66% depending on the criteria used in the study [23,24]. It has been reported that an estimated 20% of adults develop AKI while being treated in a hospital [25]. It should be stressed that AKI is often caused by renal ischemia/reperfusion [26].

Cardiovascular and cerebrovascular diseases frequently coexist with renal disease [27]. Cardio-renal syndromes, for example, often exhibit a bidirectional relationship between the heart and kidneys. Cardiac dysfunction and failure can contribute to concurrent AKI and CKD and vice versa, creating a detrimental cycle [24]. Hypertension is the leading cause of end-stage renal disease (ESKD) [6].

Diabetes mellitus (DM) represents another serious burden in association with kidney disease, with 20–40% of DM patients presenting with co-existing CKD [28,29]. DM is the leading cause of CKD and one of the leading causes of ESKD [6,30]. It is also commonly interlinked with cardiovascular disease in addition to kidney disease. Overall, the prevalence of each condition contributes to such high disease burden. Diabetes has been reported to be the most common cause of kidney failure globally [30,31,32]. Risk factors for patients with DM to progress to CKD include aging, smoking, obesity, and hypertension [33].

Cancer patients are also impacted by high disease burden in association with kidney injury. Cisplatin is a chemotherapeutic used in breast, ovary, testis, head, lung, and neck cancer [20]. Cisplatin-induced acute kidney injury (CI-AKI) has been reported to be a major comorbidity. A reported estimate of 30% of patients who receive cisplatin potentially present with AKI after one high dose, increasing the risk of CKD [20,34]. Its wide use stands in contrast to its severe limitations as an efficacious treatment, demonstrating the additional burden of preventing and treating its nephrotoxic side effects.

In addition to concurrent illnesses, treatments performed during extended hospital stays can also increase risk of kidney damage [25], including but not limited to postoperative renal failure, contrast-associated acute kidney injury (CA-AKI), cardiac-surgery-associated acute kidney injury (CSA-AKI), and sepsis-associated acute kidney injury (SA-AKI) [11,35,36].

### 1.2. Kidney Structure, Function, and Clinical Significance in Kidney Disease

The kidneys are vital organs whose proper function plays a key role in regulating homeostasis. Waste and foreign products such as urea, uric acid, creatinine, drugs, and toxins are eliminated while necessary substances such as proteins and glucose are retained [37]. Other central functions include regulation of chemical homeostasis, hormone concentrations, blood pressure through the renin–angiotensin–aldosterone system, erythropoiesis, calcium balance, oxygen sensing, cytokine homeostasis, and endothelial signaling [6]. Kidney disease can lead to loss of these functions.

Dysfunction in the kidneys can hamper waste elimination, which can manifest as AKI or CKD. Measures of estimated GFR and albuminuria, such as the albumin-to-creatinine ratio, can be used to diagnose AKI and CKD. Kidney Disease Improving Global Outcomes (KDIGO) consensus definitions use serum creatinine measurement, a surrogate for GFR [38], and urine volume as clinical criteria to identify AKI. AKI is diagnosed when increases in serum creatinine or decreases in urine volume are measured or observed, typically within a relatively short time window [39].

Transition to CKD, on the other hand, is characterized by persistent renal structural damage. KDIGO defines CKD as presence of kidney structure abnormality and function, present for more than three months, and with implications for health. The definition further classifies CKD according to its cause, glomerular filtration rate, and albuminuria categories. A diagnosis of diabetic kidney disease (DKD) is advised in patients with type 1 or type 2 diabetes and who exhibit clinical evidence of CKD attributable to diabetes [40]. However, several studies suggest that these definitions do not account for phenotypic differences which may influence earlier detection and appropriate, individualized treatment for precision medicine [41,42]. Nonetheless, these definitions highlight the close relationship between structural dysfunction, kidney disease, and the necessity for treatments which target the precise molecular mechanisms responsible.

### 1.3. Oxidative Stress and Inflammation in Kidney Disease

Oxidative stress and inflammation are intimately linked in kidney disease from development to progression [43]. As oxidative stress increases, inflammatory responses are amplified and vice versa; redox imbalance due to oxidative stress promotes nuclear translocation of inflammatory transcription factors, while inflammatory cells generate ROS [44].

Mitochondrial toxicity through excess ROS generation is a particular vulnerability of proximal tubules. ROS play important roles in both normal cellular function and signaling, as well as pathology of disease [45,46]. Normally, ROS are balanced by the defensive activity of antioxidants. However, imbalances in the production of ROS and antioxidant abundance can lead to oxidative stress [14]. ROS damage is a major contributor to oxidative stress and can trigger pathophysiological cascades of events resulting in lipid peroxidation, impaired tubular transport and cellular function, mitochondrial dysfunction, and cell death [47].

Renal ischemia represents a context for mitochondrial dysfunction to contribute to kidney injury. Mitochondrial dynamics, essential for maintaining mitochondrial homeostasis and energy production, become impaired during renal ischemia. Disruption to mitochondrial dynamics may result in morphological damage and fragmentation of mitochondria and thus lead to tubular cell damage and death. Excessive damage to mitochondria under these conditions can elevate ROS levels, precede oxidative stress, and promote apoptosis of proximal tubular cells [48,49].

Diabetes, as mentioned earlier, is the leading cause of CKD, which eventually can progress to end-stage renal failure (ESRF). Diabetes is characterized by hyperglycemia, a known disruptor of redox balance and risk factor for CKD [33]. Mitochondrial dynamic dysfunction has been proposed as an underlying mechanism driving DKD progression [50]. Diabetes has been reported to directly damage renal tubules and result in proximal tubule hypertrophy, hyper-reabsorption, and hyperfiltration [2,51]. Several producers of ROS play a role in DKD pathogenesis. Reduced citric acid cycle products and electron transport chain leakage provide sources of excessive ROS. Expression of numerous NADPH oxidase (NOX) isoforms is elevated in hyperglycemia, producing ROS as a byproduct of their enzymatic activity [52,53]. NOX proteins normally function as regulators of glucose metabolism and in the insulin signaling cascade [46]. However, excess NOX2 and NOX4 activation has been observed in experimental mouse models of diabetes. The proximal tubules contain sodium–glucose cotransporter 2 (SGLT2), which reabsorbs most glucose filtered by the glomerulus, making proximal tubular epithelial cells notably susceptible to ROS damage caused by hyperglycemia in diabetes and associated diabetic nephropathies [54].

The proximal tubules are the major site of the nephron where cellular uptake and accumulation of cisplatin occur. Uptake is reported to be mediated by the receptors Crt1 and OCT2 (copper transporter 1 and organic cation transporter 2) [20]. One mechanism of nephrotoxicity in AKI induced by cisplatin involves the proximal tubules undergoing acute tubular necrosis mediated by oxidative stress. While the exact mechanism remains to be elucidated, administration of low-dosage cisplatin has been extensively reported to cause intrastrand DNA crosslinks, impairing crucial DNA repair mechanisms, inhibiting viable DNA replication template production, and stimulating cell-cycle arrest leading to cell death. Cisplatin also preferentially targets intracellular mitochondrial DNA as it accumulates to form mtDNA adducts. The damage caused by such interaction provides a direct link to elevated ROS levels. As repair mechanisms decline and proinflammatory pathways such as NF-κB activate, renal injury caused by cisplatin culminates in renal fibrosis, afferent arteriole vasoconstriction, subsequent decline of GFR, and cell death [55]. ROS involved in cisplatin-induced multiple organ failure activate an overwhelming amount of diverse cytokines due to proinflammatory pathway induction, such as tumor necrosis factor-alpha (TNF-α), interleukin (IL)-6, and IL-1β [56].

Cadmium is another nonessential heavy metal that accumulates in the proximal tubule to induce ROS generation through mitochondrial interaction. Several studies have shown that cadmium-induced oxidative renal damage occurs through mitochondrial uptake and accumulation. Non-toxic cadmium–metallothionein complexes are transported through the blood for filtration at the glomerulus and reabsorption by proximal tubular cells. As metallothionein stores deplete, cadmium accumulates in the proximal tubule, allowing for cellular uptake, including by mitochondrial membrane channels. Excessive interaction of cadmium with mitochondrial components induces oxidative damage to renal structures by disrupting redox balance in mitochondria. This culminates in mitochondrial deformation, metabolic dysfunction, and overall elevated ROS, driving necrosis, apoptosis, and kidney injury [14,57].

Another major driver of kidney disease is the activation of inflammatory pathways [58,59]. Inflammation is associated with both AKI and CKD and is present in the transition from AKI to CKD [60]. The immune cells involved in renal immune responses mediate both pathogenic and protective mechanisms. Persistent proinflammatory responses due to stress in the kidneys can lead to progressive renal fibrosis, characteristic of CKD [61]. Innate immunity relates directly to AKI pathogenesis, as evidence has shown in animal models [62,63,64]. In addition to locally released proinflammatory cytokines such as IL-6, TNF-α, and IL-1β, systemic inflammation can hamper recovery significantly when levels of cytokines are elevated in AKI, increasing risk of CKD [43].

A diverse range of immune cells participate and play central roles in immune mechanisms, including monocytes, macrophages, neutrophils, dendritic cells, lymphocytes, and regulatory T-cells. Regeneration of damaged cells in the proximal tubules requires repair mechanisms to respond to necrosis. Macrophages and dendritic cells express Toll-like receptors 2 (TLR2) and 4 (TLR4) to recognize endogenous ligands released by damaged tubular cells [63,64]. Evidence shows that important steps in neutrophil recruitment can be inhibited in AKI and are unresponsive to treatment, weakening defenses and regeneration in sepsis-related kidney disease. Moreover, maladaptive repair mechanisms in kidney disease can progress to CKD [65].

Additionally, the innate immune response is activated concurrently with oxidative stress when endothelial or tubular cells sustain injury. Excessive ROS generation is a driver of active inflammatory pathways, bridging both mechanisms to kidney disease progression. Inflammation in AKI activates the innate immune receptors such as Toll-like receptors (TLR), Nod-like receptors (NLR), C-type lectin receptors (CLRs), and retinoic acid-inducible gene (RIG) I-like receptors to detect pathogen-associated molecular patterns (PAMPs) and damage-associated molecular patterns (DAMPs). Downstream of their activation is the nuclear factor κB (NF-κB) pathway, amongst others [66].

### 1.4. Nrf2 and NF-κB Systems in Kidney Disease

Nrf2 and NF-κB are important transcriptional regulators of oxidative stress and inflammation. The Nrf2–Keap1 system regulates the antioxidant defense system, which endogenously scavenges ROS generated during oxidative stress [67], while NF-κB, as alluded to, is activated to regulate expression of proinflammatory mediators [68]. Just as oxidative stress and inflammation are linked, Nrf2 and NF-κB pathways are also tied together in the redox balance of renal cells.

Nrf2, nuclear factor erythroid 2-related factor 2, is a ubiquitous transcription factor and is considered a master regulator of oxidative stress [69]. It exists in the cytoplasm as a complex, inactivated by a bound adaptor protein Kelch-like ECH-associated protein 1, Keap1. Under normal conditions, Nrf2 basal levels are kept low through negative regulation by Keap1-facilitated ubiquitination. When ROS are high, key structural modifications to Keap1 cysteine residues relieve Nrf2 binding, thus allowing it to translocate to the nucleus and bind to its target sequence, the antioxidant response element (ARE) promoter. In addition, a Keap1-independent degradation pathway as well as other kinases have the potential to regulate the activity of Nrf2 [69,70]. Once bound, Nrf2 promotes expression of a wide range of cytoprotective genes, including HMOX1 encoding the heme oxygenase-1 (HO-1) enzyme [70]. HO-1, a downstream target of Nrf2, plays a key role in the oxidative stress response, preventing Nrf2 from being degraded and protecting mitochondria in human embryonic kidney cells. It has also been suggested as an AKI candidate biomarker. It plays a protective role in kidney injury by promoting anti-inflammatory antioxidants [71]. Nrf2 has been implicated as a critical component to attenuate CKD-related oxidative damage [70]. It should be pointed out that Nrf2 activation in kidney injury could, in certain circumstances, further aggravate kidney injury; therefore, a need to understand the negative effect of Nrf2 activation in the context of kidney injury is warranted [70,72,73,74].

NF-κB, nuclear factor kappa B, is also a transcription factor. NF-κB is an important pathway for regulation of proinflammatory cytokine and chemokine secretion, as well as cell survival and growth. NF-κB translocates to the nucleus to affect gene expression upon induction by proinflammatory cytokines, TLR signaling, oxidative stress, and ischemia/reperfusion [26]. In the canonical pathway, NF-κB transcription factors remain inactive in the cytoplasm as a complex. Oxidative stress generates ROS, activating Ikϐ kinase (IKK) to phosphorylate inhibitory NF-κB pathway proteins. Negative regulators such as Ikϐ are subsequently removed via ubiquitination [44,46]. The renin–angiotensin system is also activated and generates ROS in CKD, elevating levels of NF-κB and promoting proinflammatory cytokines TNF-α, IL-1, IL-6, IL-2, and MCP-1 [70].

Nrf2 and NF-κB are linked mechanistically. NF-κB binding sites on the Nrf2 gene allow for induction of the Nrf2–Keap1 pathway upon interaction with inflammatory elements. Nrf2 can suppress NF-κB in turn to reduce transcriptional activity and thus limit its inflammatory potential [70]. Indirect modulation occurs bidirectionally as well. NF-κB is capable of Nrf2 transcriptional inhibition by its p65 subunit and inactivation through competitive inhibition, while Nrf2 can be activated by anti-inflammatory compounds through interference with NF-κB activation. Furthermore, HO-1 can inhibit NF-κB transcription [69]. In an IRI mouse model, inhibition of Nrf2 showed an elevated activation of NF-κB and activated M1 macrophage polarization, exacerbating proinflammatory agent release including TNF-α and iNOS (inducible nitric oxide synthase). Nrf2 inhibited M1 macrophage polarization by antagonizing NF-κB signaling and thus ameliorating renal IRI [75]. Balance between the two systems is linked to the context of their activation. It is important to note that homeostasis relies on the coordinated activation of both Nrf2 and NF-κB to regulate antioxidant and inflammatory responses. In kidney disease, this balance is critically disrupted, shifting towards maladaptive repair mechanisms, cellular injury and death, and culminating in fibrosis if untreated.

**Figure 1:**
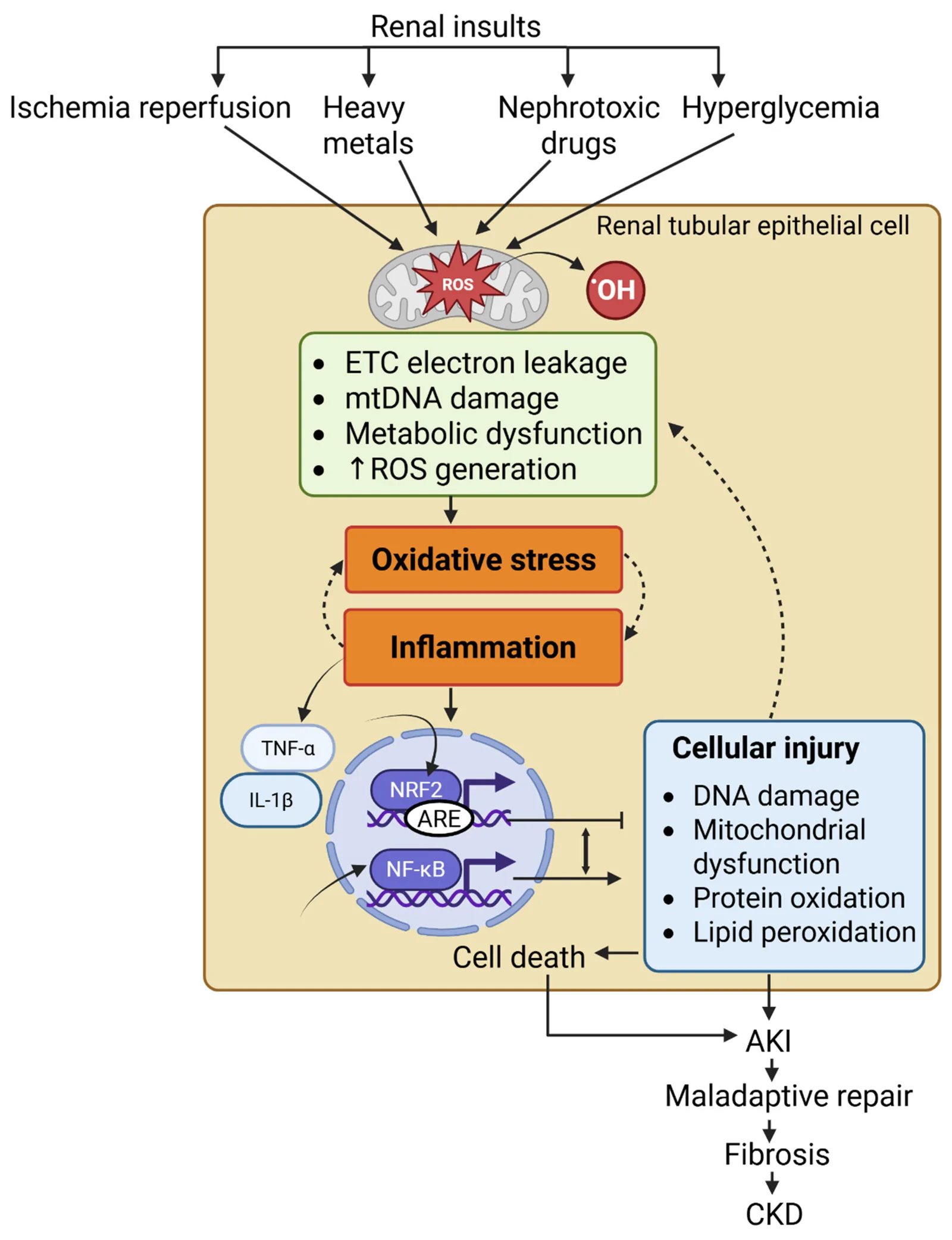
Renal tubular epithelial cells are targets for renal insults including IRI, heavy metals, nephrotoxic drugs such as cisplatin, and hyperglycemia. These insults can induce ROS generation, metabolic dysfunction, mitochondrial DNA damage, and electron leakage from the electron transport chain. Oxidative and inflammatory stress responses are activated and fuel a cycle of further ROS generation and release of proinflammatory cytokines such as TNF-α and IL-1β. Transcription factors NRF2 and NF-κB translocate to the nucleus from the cytoplasm, where their respective pathways crosstalk to modulate oxidative stress and inflammatory responses to DNA damage, mitochondrial dysfunction, protein oxidation, and lipid peroxidation. Extensive injury promotes renal tubular epithelial cell death and potential contribution to AKI. Persistent activation of NF-κB may promote renal fibrosis and eventually CKD if left unmanaged.

In relation to kidney disease, there is evidence of Nrf2 and NF-κB as potential therapeutic targets. It has been reported that NRF2 activity decreased in CKD animal models with increased intrarenal ROS generation and oxidative stress, which also exhibited increased activation of NF-κB and immune cell infiltration [76]. In renal IRI, a positive feedback loop between disease-induced TNF-α production and subsequent activation of NF-κB by TNF-α highlights a key source for inflammatory injury to renal cells [26]. The Nrf2 activator and NF-κB inhibitor bardoxolone methyl was shown to induce Nrf2 and suppress NF-κB in renal tubular cells. Cisplatin has been shown to increase expression of NF-κB and TNF-α in the kidneys [77]. In cisplatin-induced kidney disease, a chemotherapeutic, celastrol, suppressed NF-κB to ameliorate nephrotoxicity and preserve mitochondrial integrity [69]. Other compounds have been explored for protective Nrf2-system responses in kidney disease [70]. These findings support investigation of potent activators of oxidative stress and inflammation modulators in kidney disease [44] (Figure 1). Edaravone, a potent antioxidant and anti-inflammatory compound, may be of interest in this context.

## 2. Edaravone: Pharmacology and Mechanism of Action

### 2.1. Brief History of Edaravone Clinical Use

**Figure 2:**
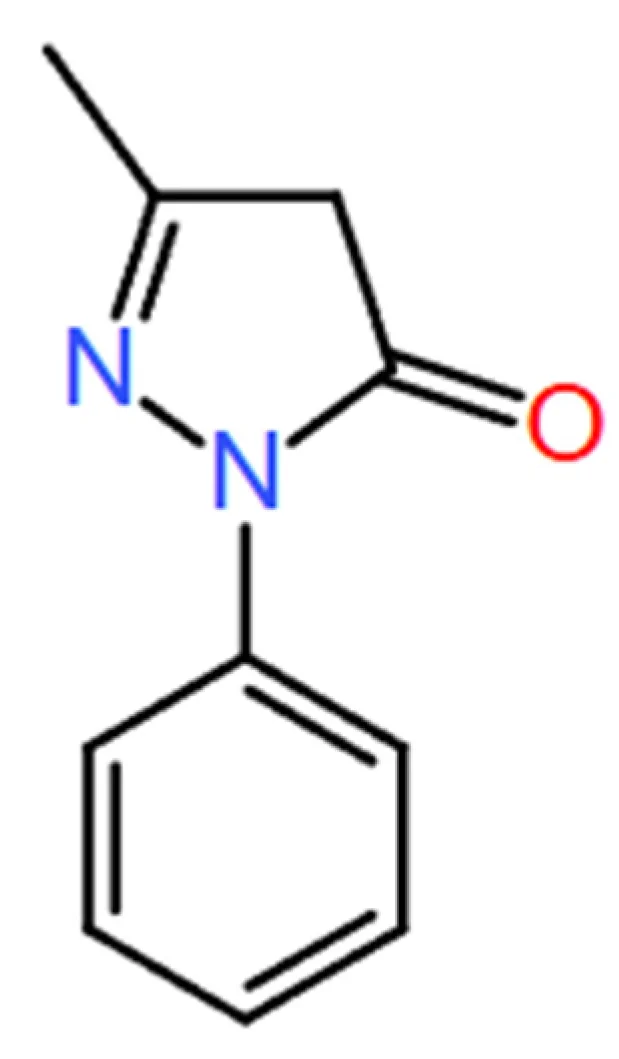
The chemical structure of edaravone. The FDA approved its use in the US for treatment of ALS in May 2017. Evidence of its safety and efficacy was supported by the phase 3 MCI186-19 (Study 19) trial, which showed slowed functional decline in well-defined ALS patients in Japan being treated with IV EDA [87]. The physiochemical properties of EDA limited its administration to IV and injection, in part due to poor bioavailability and increased patient compliance. Advances in formulation subsequently allowed for development of the oral form [88]. It has been marketed as Radicava^©^ and Radicava ORS^©^ and is available in both IV and oral forms, respectively. In Japan, oral use of the drug for ALS was approved quite recently in December 2022 [89].

Edaravone (EDA, 3-methyl-1-phenyl-2-pyrazolin-5-one) (Figure 2), known as Radicut^©^ in Japan, is an effective, synthetic free-radical scavenger [78]. Clinical studies began in 1987, with EDA under the code name of MCI-186 [79]. EDA was introduced into clinical use in 2001 after initially being developed by Mitsubishi Yuka Pharmaceutical Co/Mitsubishi Chemical Industries. Mitsubishi Tokyo Pharmaceuticals Co obtained approval for manufacture in 2001 [80], while Mitsubishi Tanabe Pharma Corp currently markets the drug [81]. Japan was the first to approve EDA as a treatment for acute ischemic stroke (AIS), administered intravenously (IV). It is marketed in China as Yi Da Sheng^©^, Youmin^©^, and Bicun^®^ [78]. Its designated status as an orphan drug for ALS in 2005 supported its development for other serious, neurodegenerative conditions. In 2015, use of EDA to treat ALS was approved for use in Japan and South Korea [82]. For treatment of ALS patients, EDA was given at 60 mg IV once daily for 14 days for the first cycle, and it was determined that the terminal half-life of EDA was 4.5–6 h with 70–90% urinary elimination via sulfate/glucuronide conjugation metabolism [83]. The 2021 Japanese Stroke clinical guidelines continue to recommend EDA as a treatment for acute ischemic stroke for its neuroprotective properties [84]. Subsequently in China, an IV combination therapy of edaravone dexborneol was approved in 2020 as a brain cytoprotective agent for improved AIS patient outcomes [85,86]. EDA is also marketed by Edinburgh Pharmaceuticals as Arone^©^, Nuravon^©^, and Fraseda^©^ in India [87].

### 2.2. Structure and Pharmacological Properties

EDA is a five-membered heterocyclic pyrazolone derivative with a low molecular weight of 174.2. EDA acts as a free-radical scavenger to neutralize ROS, inhibits both hydroxyl-dependent and -independent lipid peroxidation, and prevents damage caused by oxidative stress [90]. EDA is lipophilic and can cross the blood–brain barrier, a key feature in its ability to protect nerve cells and vascular endothelial cells from the damaging effects of oxidative stress and inflammation [91].

### 2.3. Free-Radical-Scavenging Mechanisms of EDA

As described above, renal injury can be caused by excess ROS generation. Oxidative damage leads to cellular damage through lipid peroxidation of membranes [92], protein damage [93], and DNA damage [94], all of which may result in cell death. EDA may mitigate the effects of oxidative stress through its antioxidant mechanisms by directly scavenging reactive radical species and inhibiting lipid peroxidation [80].

EDA demonstrates acid–base dissociation and keto–enol tautomerism. This tautomerism leads to an equilibrium between molecular structures, including neutral and anionic keto, enol, and amine forms, which underlie its relatively broad radical-scavenging ability [80].

EDA exists as mixtures of neutral and anionic forms, the latter of which is particularly effective against certain radicals and is more than three times more abundant at physiological pH, reported to be 28.5% for the neutral form and 71.5% for the potentially more reactive anionic form, as supported by several studies [95,96]. EDA targets oxygen-derived free-radical species, showing high scavenging abilities against hydroxyl radical, tert-butylperoxyl radical, and methyl radical compared to other radical scavengers such as uric acid, glutathione, and trolox [97].

EDA’s key mechanism of action is direct radical scavenging through hydrogen atom transfer (HAT), single electron transfer (SET), and radical adduct formation (RAF). SET has been shown to contribute most to overall hydroxyl radical reactivity in its anionic form, while RAF was found to be the main mechanism for the neutral form. Overall, SET is the initial step of the two-step mechanism, sequential electron–proton transfer (SEPT), which predominates [80].

Hydroxyl and lipid peroxyl radicals are good targets for EDA, as hydroxyl radicals indiscriminately oxidize cellular components while peroxyl radicals oxidize lipids, extracting hydrogen atoms from neighboring fatty-acid side chains to begin the propagation stage of lipid peroxidation. In addition, EDA reacts with a variety of species, including alkoxyl radicals, superoxide anions, peroxynitrite, and nitric oxide [98].

Some other suggested mechanisms of action of EDA include prevention of ferroptosis, H_2_O_2_ scavenging by peroxiredoxin-2, restoration of mitochondrial membrane potential and ATP through Nrf2, and restoration of stability of profilin-1 (PFN-1) [79].

It should be pointed out that redox-sensitive pathways are particularly relevant to the ability of EDA to neutralize a broad range of ROS [99]. As ROS are upstream of endogenous antioxidant systems, an excessive level may overwhelm defenses and contribute to dysregulated protective responses while simultaneously allowing proinflammatory signals to continue unchecked. By inhibiting ROS and lipid peroxidation upstream, EDA may address both oxidative stress and inflammation stimuli to bolster radical defense while reducing cytokine-mediated renal damage.

Nevertheless, one question arising here is why prior antioxidant trials (such as vitamin E and N-acetylcysteine) in kidney injury largely failed. The reason for this is likely multifactorial, including mechanism breadth, timing, and lipophilicity, which may differentiate EDA from these antioxidants. In this respect, an excellent contrast-induced nephropathy study bridging EDA’s preclinical and clinical aspects was published in 2024 in which post-procedure serum creatinine showed a significant drop [100], demonstrating the clinical effectiveness of EDA. Nonetheless, the FDA’s warning regarding EDA’s use along with sodium bisulfide excipients should be taken into account as hypersensitivity, anaphylaxis, and asthma may compromise EDA’s efficacy.

### 2.4. Anti-Necrotic, Anti-Inflammatory, Anti-Apoptotic, and Cytoprotective Effects of EDA in Renal Animal Models

Animal models have been useful in observing a range of EDA’s ameliorative effects on renal disease damage in addition to its direct free-radical-scavenging ability. Renal ischemia–reperfusion injury (IRI) provides a well-established context in which lipid peroxidation, mitochondrial dysfunction, oxidative stress, inflammation, and cell death can be studied to further investigate AKI and CKD. Nephrotoxic models were also found to benefit from EDA administration.

In an early rat model study, EDA was found to scavenge ROS produced in the renal tubular cells, reducing ROS levels and inhibiting lipid peroxidation of cellular and subcellular membranes in vivo. A decrease in ROS production was also observed in vitro in a dose- and time-dependent manner [101]. ROS-mediated lipid peroxidation contribution to renal ischemic injury through cellular and mitochondrial membrane damage can thus be reduced by EDA’s inhibition. Additional evidence points to mechanisms of EDA to ameliorate endogenous antioxidant systems in IRI models. Lipid peroxidation was found to be inhibited in a subsequent rat study investigating the effects of EDA on IRI-induced oxidative stress. Mitochondrial superoxide dismutase (MnSOD/SOD2), an endogenous antioxidant enzyme targeting superoxide, was upregulated. This study also incorporated a nitric-oxide-donor moiety into EDA, enhancing its anti-inflammatory benefits through reduced NF-κB nuclear translocation after renal IRI [102]. This finding provides more evidence of NF-κB involvement within renal pathogenesis.

Mitochondrial protection and suppression of cell death can also be examined in experimental models. In another rat study involving IR-induced kidney injury, EDA inhibited apoptosis in kidney tubular epithelial cells, reduced mitochondrial injury, and prevented apoptosis and necrosis through suppression of the JAK/STAT pathway. JAK/STAT is reported to promote development and progression of renal disease, making it a suitable target for drug treatments. JAK/STAT mediates apoptosis, inflammation, and immune regulation, among other processes [103]. Its activation is associated with oxidative stress by increasing ROS generation and cell death. EDA’s suppression of JAK/STAT-mediated ROS and cytokine release can be credited to its anti-necrotic, anti-apoptotic, and anti-cytokine effects [104]. It should be noted that the JAK/STAT system has three family members—JAK1, JAK2, and STAT3—each of which plays a distinct role in kidney injury [105,106,107,108,109,110]. However, only one study could be found in the literature in terms of EDA’s effect on the JAK/STAT signaling pathways in ischemic AKI, potentially overemphasizing the protective role of EDA in this pathway.

Further evidence recently reported comes from connections to the Nrf2 pathway. A rat model of unilateral ureteral obstruction investigating the effect of EDA and EDA in combination with sorafenib, a multi-kinase inhibitor approved for treatment of kidney cancer, found a significant increase in Nrf2/HO-1 expression in renal cells and a significant decrease in caspase-8 expression [111]. This was in conjunction with a decrease in markers of oxidative stress, inflammation, such as TNF-α and IL-1β, apoptosis, and renal fibrosis, supporting the anti-inflammatory, anti-apoptotic, and oxidative-stress-inhibiting effect of EDA [111]. It should be noted that the anti-fibrotic effect of EDA has also been reported in other animal models of kidney injury [112,113].

In a rat study by Wan et al., renal-artery-injected siRNA targeting IKKβ was shown to prevent renal IRI and inhibit activation of NF-κB. A reduction in inflammation and renal injury was observed through inhibition of binding between NF-κB and DNA and reduced expression of AKI markers neutrophil gelatinase-associated lipocalin (NGAL) and IL-8 [114]. Inhibition of IKKβ is associated with reduced renal injury and represents a therapeutic target which may be affected by upstream inhibition of inflammatory activation by EDA.

Cisplatin-induced renal dysfunction in rats was found to be reversed after repeated injection with EDA, as creatinine levels and blood urea nitrogen were reduced significantly. Near-complete renal protection was reported and continued to be observed one day after cisplatin injection while reversing glutathione depletion. Taken together, this finding suggests that EDA preserves endogenous antioxidant defense post-injury to protect renal tubules, particularly as histological changes such as vacuolization, necrosis, and protein casts were reversed as well [115,116].

**Figure 3:**
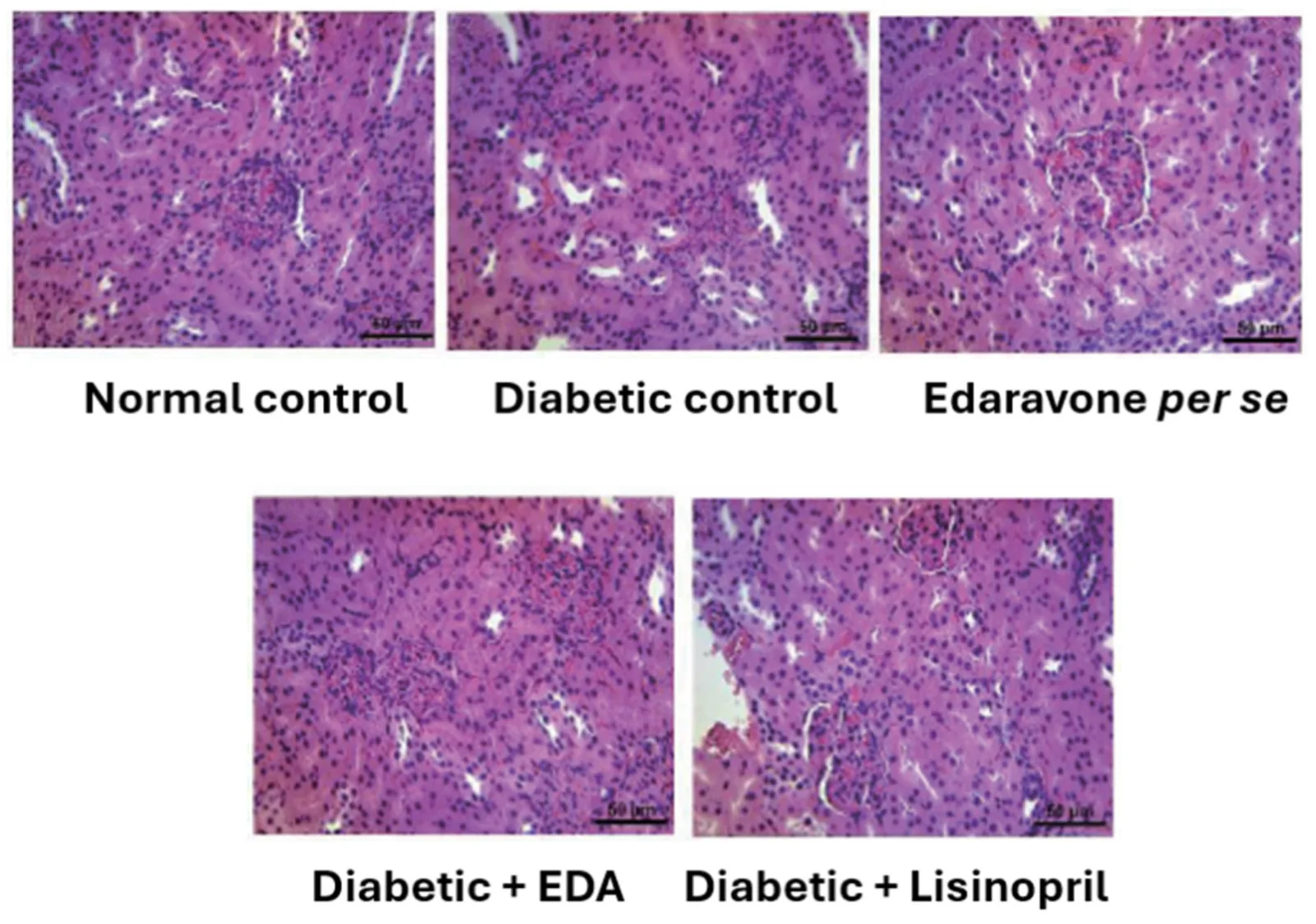
Protective effects of edaravone on renal histology stained by hematoxylin and eosin. This figure was reproduced from Varatharajan et al. [117].

**Figure 4:**
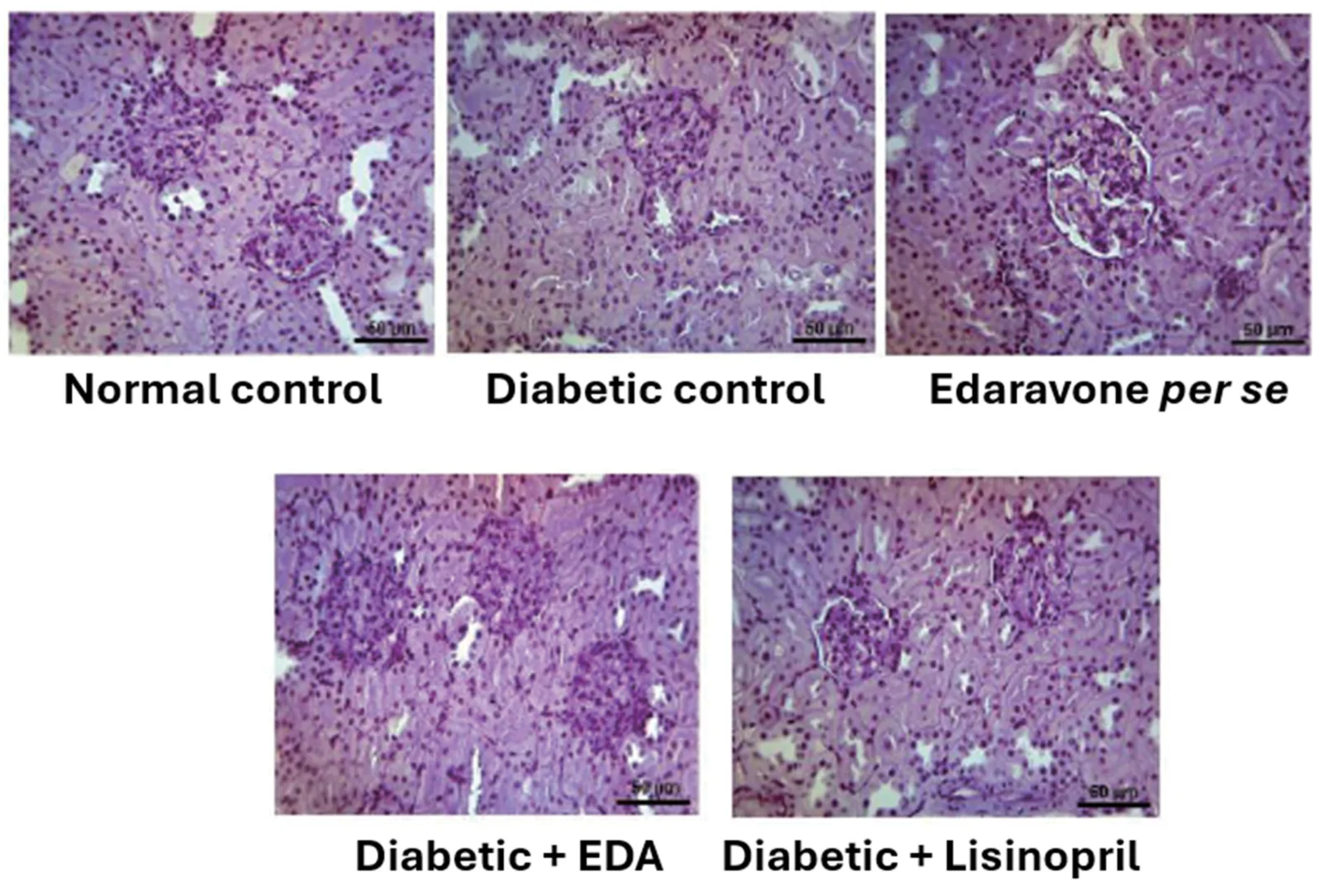
Protective effects of edaravone on renal histology stained by periodic acid–Schiff. This figure was reproduced from Varatharajan et al. [117].

The protective effect of EDA on DKD has also been investigated. Using diabetic rats induced by streptozotocin (STZ), Varatharajan et al. [117] have demonstrated that diabetes-induced renal abnormalities could be markedly mitigated by EDA as shown in Figure 3 and Figure 4, in which both hematoxylin/eosin staining and periodic acid–Schiff staining were performed to visualize kidney histology. This mitigation is in conjunction with decreased levels of serum creatinine and blood urea nitrogen. While EDA has no effect on blood glucose levels, its renoprotective effect is like that of lisinopril, which is known for its kidney-protective power in diabetes [118]. It should be noted that the animal model used in this study is that of type 1 diabetes; therefore, more studies regarding EDA’s renoprotective effects remain to be carried out using animal models of type 2 diabetes that may be induced by a variety of approaches [119].

**Figure 5:**
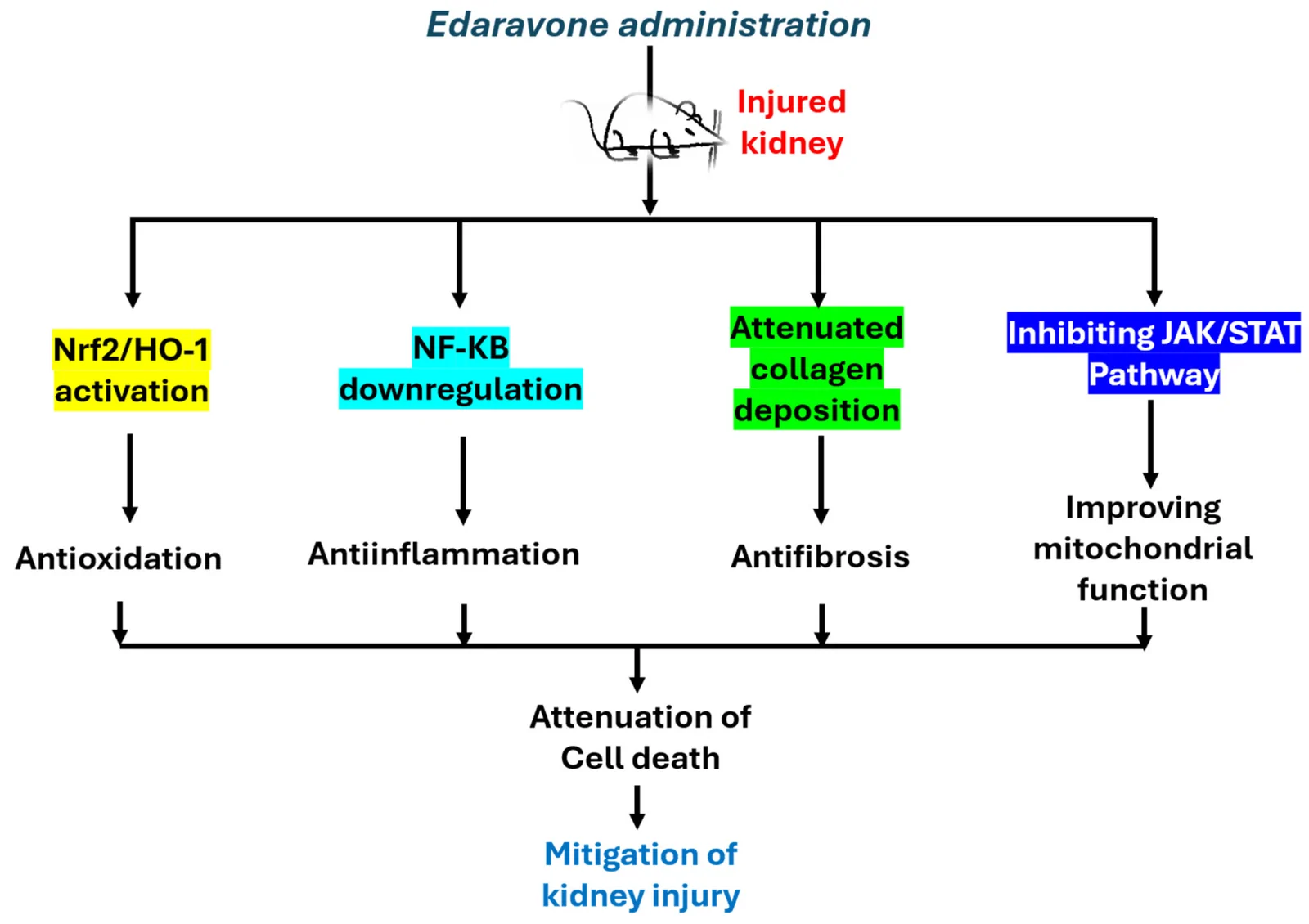
Mechanisms by which edaravone protects the kidney against a variety of renal injuries in animal models.

**Figure 6:**
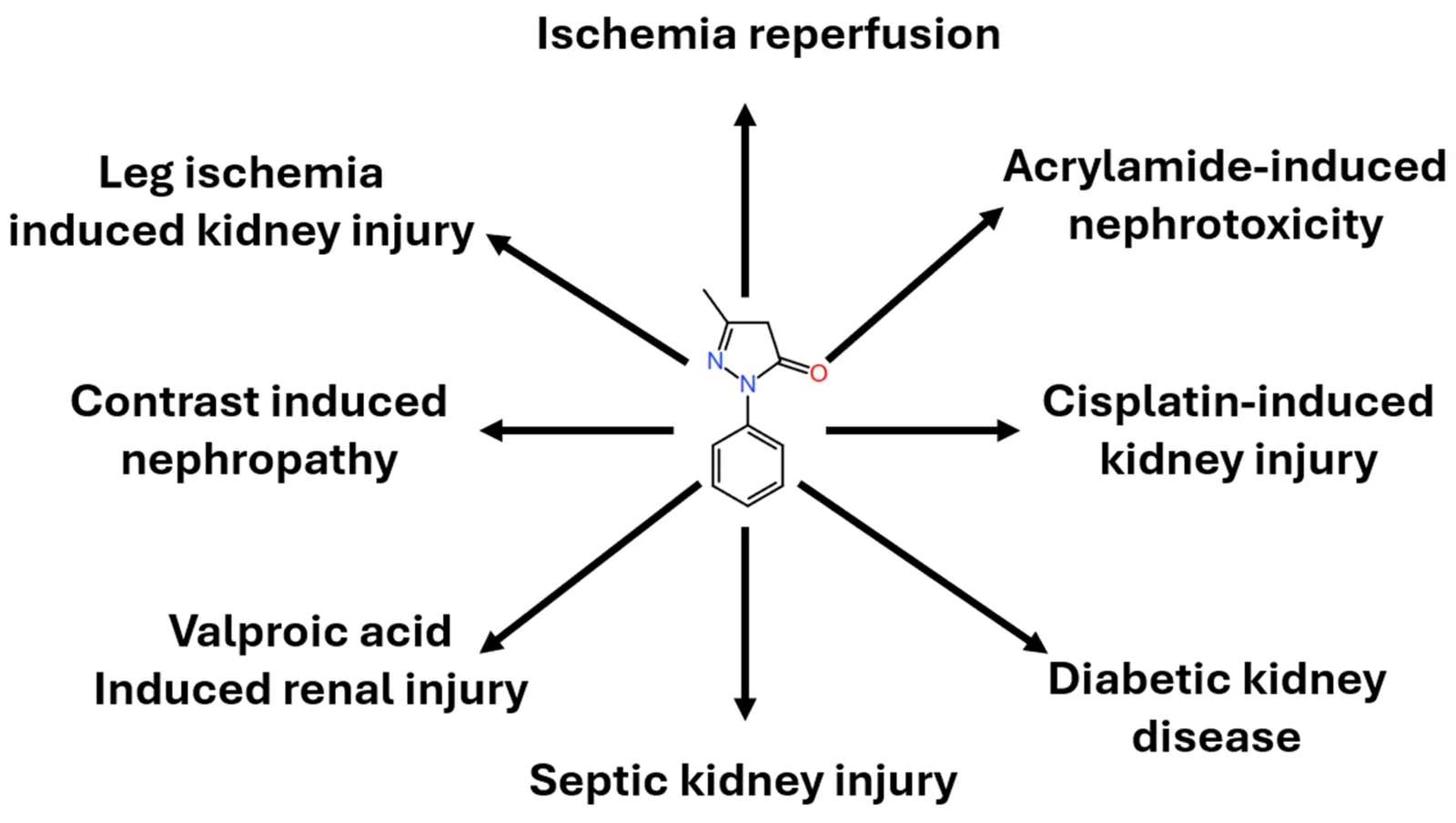
Animal models of kidney injury used to assess the renoprotective effects of edaravone.

In summary, as shown in Figure 5, animal model studies suggest the potent effects of EDA may extend beyond direct scavenging of ROS. Inhibition of lipid peroxidation, increased expression of endogenous antioxidant defenses, suppressed inflammatory signaling, mitochondrial protection, and decreases in apoptosis, necrosis, and fibrosis-related tubular injury have been reported. Limited progression of AKI to CKD and other renal injury by EDA can thus be supported by animal models, as summarized in Figure 6, which include renal injuries induced by ischemia/reperfusion, acrylamide [120], cisplatin [19], diabetes [117], sepsis [121,122], valproic acid [123], contrast [124], and leg remote ischemia [125]. Many of these animal model studies show EDA’s common underlying renoprotective mechanisms as outlined in Figure 5.

### 2.5. Cautions

It should be noted that ALS clinical guidelines in Japan mention contraindication of EDA with severe renal dysfunction, indicating cautious use for those with AKI and CKD. This is also mentioned in the FDA’s medical review of EDA based on Study 19. While it was noted that no strong safety signals were detected, renal failure was not definitively ruled out. It is worth investigating EDA in patients with renal disease for safety and efficacy. Therefore, despite promise in other diseases and experimental models, cautions must be considered, as few adverse events involving the kidneys have been reported in patients. Toxicity of EDA may be related to redox capacity of generated hydroxylated derivatives, although no established mechanism has been proposed to suggest this as a definitive connection found in clinical evidence of EDA-induced renal injury [126]. It should be noted that whether the aforementioned dosage, route of administration, and renal metabolic profile of EDA [83] can also apply in treating patients having kidney dysfunction remains to be assessed.

In the broader context of its clinical safety record, EDA has a promising safety profile. In post-market surveillance of EDA, where no factor beyond the drug was detected as a possible cause, 17 patients (8.2%) had renal disorders. However, in the remaining patients reported to have renal disorders, other factors could potentially be attributable [127]. Moreover, a phase I clinical study of EDA in healthy Chinese volunteers found a parenteral solution of EDA to be both well tolerated and safe in several dose regimens [128]. Another study noted that in the 17 years of clinical experience with EDA to that point in 2010, few adverse events were noted [81]. Indeed, as recently as 2026, it has been noted that IV EDA has a favorable safety profile [129]. New AE data will be generated for the current oral suspension rather than applying IV-administration-related AEs. To date, no animal studies have supported nephrotoxicity of EDA. Additional clinical studies are still needed to fully elucidate the pharmacokinetics, efficacy, safety, and benefits of EDA for prevention and treatment of human kidney disease. Of note, many studies regarding post-market effects have been conducted internationally [79]. Moreover, whether overdose of EDA could induce reductive stress damage in the kidneys [130,131] also remains to be evaluated, at least in rodent models. It should also be pointed out that studies cited in this review article have limitations, which include small animal studies, heterogeneous models with varying regimens, limited replications, lack of renal pharmacokinetics and controlled human trials as well as clinical dose equivalence.

## 3. Conclusions

In conclusion, edaravone is a potent free-radical scavenger used in the treatment of several disorders globally. Its therapeutic potential as an antioxidant in the context of kidney disease makes it an attractive candidate, as AKI and CKD are characterized in part by oxidative stress and inflammatory injury to renal cells. The mechanisms that drive pathogenic progression of kidney disease are still not well understood. While animal models and related clinical trials support the argument that EDA can mitigate the effects of ROS-mediated damage both directly and indirectly, further study is needed to understand the exact mechanisms by which EDA acts in kidney disease.

## Data Availability

No new data were created or analyzed in this study. Data sharing is not applicable to this article.
